# Interleukin-17A mRNA and protein expression within cells from the human bronchoalveolar space after exposure to organic dust

**DOI:** 10.1186/1465-9921-6-44

**Published:** 2005-05-25

**Authors:** Stefan Ivanov, Lena Palmberg, Per Venge, Kjell Larsson, Anders Lindén

**Affiliations:** 1 Lung Pharmacology Group, Department of Respiratory Medicine and Allergy, Institute for Internal Medicine, Göteborg University, Guldhedsgatan 10A, 413 46 Gothenburg, Sweden; 2 Lung and Allergy Research, the Institute of Environmental Medicine, Karolinska Institute, 171 77 Stockholm, Sweden; 3 Department of Clinical Chemistry and Pharmacology, Uppsala University, 751 85 Uppsala, Sweden

## Abstract

**Background:**

In mice, the cytokine interleukin (IL)-17A causes a local accumulation of neutrophils within the bronchoalveolar space. IL-17A may thereby also contribute to an increased local proteolytic burden. In the current study, we determined whether mRNA for IL-17A is elevated and protein expression of IL-17A occurs locally in inflammatory cells within the human bronchoalveolar space during severe inflammation caused by organic dust. We also assessed the expression of the elastinolytic protease MMP-9 in this airway compartment.

**Methods:**

Six healthy, non-smoking human volunteers were exposed to organic dust in a swine confinement, a potent stimulus of neutrophil accumulation within the human bronchoalveolar space. Bronchoalveolar lavage (BAL) fluid was harvested 2 weeks before and 24 hours after the exposure and total and differential counts were conducted for inflammatory BAL cells. Messenger RNA for IL-17A was measured using reverse transcript polymerase chain reaction-enzyme linked immunoassay (RT-PCR-ELISA). Intracellular immunoreactivity (IR) for IL-17A and MMP-9, respectively, was determined in BAL cells.

**Results:**

The exposure to organic dust caused more than a forty-fold increase of mRNA for IL-17A in BAL cells. IL-17A immunoreactivity was detected mainly in BAL lymphocytes, and the number of these IL-17A expressing lymphocytes displayed an eight-fold increase, even though not statistically significant. The increase in IL-17A mRNA was associated with a substantial increase of the number of BAL neutrophils expressing MMP-9 immunoreactivity.

**Conclusion:**

Exposure to organic dust increases local IL-17A mRNA and because there is intracellular expression in BAL lymphocytes, this suggests that IL-17A protein can originate from lymphocytes within the human bronchoalveolar space. The fact that the increased IL-17A mRNA is associated with an increased number of MMP-9-expressing neutrophils is compatible with IL-17A increasing the local proteolytic burden through its neutrophil-accumulating effect.

## Introduction

Several chronic lung diseases are associated with an exaggerated accumulation and activity of neutrophils locally in the human bronchoalveolar space [1]. These lung diseases include acute, severe asthma, chronic bronchitis and chronic obstructive pulmonary disease (COPD) [1]. Neutrophils may play a pathogenic role in these diseases through the release of free oxygen radicals and by producing enzymes that increase the local proteolytic burden. Thus, via these compounds, neutrophils may contribute to pathogenic hallmarks such as non-specific bronchial hyper-responsiveness and hypersecretion, as well as to epithelial damage and tissue remodelling [1].

There is evidence that, among the proteolytic enzymes in the lungs, matrix metalloproteinase (MMP)-9 plays an important pathogenic role and this is most likely due to its capacity to cleave structural proteins such as collagens and elastin [2]. In support of this, local MMP-9 is increased in asthma and in COPD [3-8]. It is believed that neutrophils constitute an important source of MMP-9 in the lungs, even though macrophages, bronchial epithelial cells and fibroblasts may also produce MMP-9 under certain conditions [2].

Recent studies on mice *in vivo *and on human cells *in vitro *are compatible with activated T-lymphocytes contributing to the local accumulation of neutrophils in the bronchoalveolar space through the release of the cytokine interleukin (IL)-17A. However it remains unknown whether lymphocytes producing IL-17A actually reside within the bronchoalveolar space of humans [1]. In the bronchoalveolar space of mice and rats, local administration of recombinant IL-17A protein increases the number of neutrophils substantially [1,9]. Systemic pre-treatment with a neutralizing anti-IL-17A antibody attenuates endotoxin-induced neutrophil accumulation in the same compartment of mice [10]. It is likely that IL-17A causes this neutrophil accumulation in part indirectly through the induced production of neutrophil-mobilizing cytokines, such as granulocyte-macrophage colony-stimulating factor (GMC-SF), IL-6 and IL-8, in local cells, including bronchial epithelial cells, fibroblasts and smooth muscle cells [1,10]. In addition, there are now studies on mice and rats indicating that IL-17A may control the local proteolytic burden in the bronchoalveolar space *in vivo *through its impact on neutrophil accumulation [11,12].

We recently showed that organic dust in a swine confinement, a strong stimulus of neutrophilic inflammation, causes a substantial increase in the concentration of free, soluble IL-17A protein in the human bronchoalveolar space [13]. Interestingly, this increase in local IL-17A protein is associated not only with an accumulation of neutrophils but also with an accumulation of lymphocytes. The current study was undertaken to determine whether these lymphocytes can produce IL-17A in humans. The study was also undertaken to address whether production of IL-17A is associated with an increased local proteolytic burden from neutrophils. Exposure to organic dust in a swine confinement was utilised as a pro-inflammatory stimulus and intracellular expression of MMP-9 was targeted as a sign of proteolytic burden in the bronchoalveolar space of human healthy volunteers.

## Methods

### Study design

The study protocol was approved by the Ethics Committee at Karolinska Institute (Diary N. KI 95:347). Six healthy, non-smoking volunteers gave their written consent to participate in the study after receiving both written and oral information.

All the participants were non-atopic, non-asthmatic as determined by history and questionnaire. They were exposed to organic dust while weighing pigs in a swine confinement (ie. a barn) during three hours, as previously described [14-16]. The procedure incorporates exposure to aerosolised organic dust, with a concentration of approximately 24 mg dust and 1.2 μg endotoxin per m^3^, as assessed in a recent study [13]. Normal lung function and normal airway responsiveness were ascertained in all subjects prior to exposure. The subjects underwent bronchoscopy with bronchoalveolar lavage (BAL-see below) 2 weeks before and 24 hours after the exposure.

### Lung function

Spirometry was performed in accordance with the criteria of the American Thoracic Society using a low-resistance rolling-seal spirometer (OHIO model 840; Airco, Madison, WI, USA) [17]. Swedish reference values were used [18,19].

### Bronchial responsiveness

The methacholine provocation test was conducted prior to exposure to ascertain a normal airway function, as previously described [20]. Briefly, after the inhalation of the diluent (vehicle) alone, doubling concentrations of methacholine were given starting at 0,5 and ending at 32 mg/ml. The test was stopped either at a FEV_1 _decrease of 20%, compared to the value registered after the inhalation of the diluent, or after inhalation of the highest methacholine provocation (32 mg/ml). The cumulative dose causing a 20% decrease of FEV_1 _(PD_20_FEV_1_) was calculated.

### Bronchoalveolar lavage

Bronchoscopy and bronchoalveolar lavage were performed according to standard procedures previously described [13]. In short, after pre-medication with morphine-scopolamine, a flexible fibreoptic bronchoscope (Olympus Type 4B2; Olympus Optical Co. Ltd., Shinjukuku, Tokyo, Japan) was inserted through the nose under local anaesthesia with lidocaine (Xylocain R, Astra, Södertälje, Sweden). The bronchoscope was wedged in a middle lobe bronchus and a total of 250 ml of sterile saline at 37°C was instilled in 5 aliquots of 50 ml. After each instillation, the BAL suspension was gently aspirated and collected in a siliconized plastic bottle kept on ice.

After sterile filtration (70 μm filter), the BAL suspension was centrifuged (Hereus Omnifuge 300 g, 10 min, +4°C). The cell-free supernatant was kept frozen (-80°C) until analysis. The cell pellet was resuspended (phosphate-buffered saline [PBS]: 5 ml). Viability of cells was assessed using trypan blue exclusion and the total cell number (ie. concentration) was determined for each sample in a Bürker chamber. BAL cytospin samples were prepared and stained (May-Grünwald-Giemsa) and differential cell counts were performed using light microscopy (Zeiss Axiopaln 2, Carl Zeiss, Jena, Germany).

### Messenger RNA for IL-17A

Remaining cells from the resuspended pellet were centrifuged (see above) and the supernatant was removed. The cell pellet was re-suspended (PBS: 50 μl plus RNAlater from Ambion Inc, Austin, USA: 250 μl per 3 × 10^6 ^cells), transferred into microtubes, (3 × 10^6 ^cells per tube) and incubated (24 hrs, +4°C). The RNeasy minikit was used according to the manufacturer's protocol with the following modification: The eluted RNA was treated with Dnase I (Rnase-Free Dnase set: 30 min, 37°C). After this, the steps from adjusting binding conditions to eluting RNA were repeated.

The eluted total RNA was measured for quantity and purity using spectrophotometry (SpectraMaxPlus^®^Molecular Devices Corporation Sunnyvale, CA, USA; absorbance at 260 and 260/280 nm respectively) and then stored frozen (-83°C) until purification of Total RNA.

The RT-PCR ELISA technique was employed to quantify relative changes in the IL-17A mRNA gene transcript. A one-step RT-PCR was utilized with a Gene Amp PCR system 2400 (Perkin Elmer, Wellesley, MA, USA) for amplification. Each RT-PCR of 50 μl was conducted using 100 ng of total cellular RNA and 30 pmol of each primer, 10 U RNase inhibitor (recombinant RNasin, Promega Corporation, Madison, WI, USA), 200 μM PCR DIG Labelling mix (20 μM dATP, dGTP, dCTP each plus 19 μM dTTP plus 1 μM DIG-dUTP), 5 mM DDT, 10 μl RT-PCR reaction buffer (1.5 mM Mg+), 1 μl Titan enzyme mix (AMV reverse transcriptase+Taq DNA polymerase+Pwo DNA polymerase) or 0,5 μl Expand high fidelity PCR system (Taq DNA polymerase+Pwo DNA polymerase) for DNA controls (all reagents from Roche Diagnostics). Reverse transcription was performed (30 min, 50°C). The IL-17A and the house-keeping gene HPRT were subsequently annealed (55°C). IL-17A and HPRT were then amplified (35 and 30 cycles, respectively) and the DIG labelled PCR product was stored frozen (-20°C) before the detection step. RT-PCRs were performed in duplicate. Gene sequences were accessed from NCBI Database and accession number used was for human (h) HPRT V00530 and for hIL-17 U32659. Scandinavian Gene Synthesis AB (Köping, Sweden) provided the oligonucleotides.

The gene primer sequences used for RT-PCR and ELISA detection:

hHPRT

Sense (5') 5'CGT CGT GAT TAG TGA TGA TGA AC3'

Antisense (3') 5'GCA AAG TCT GCA TTG TTT TGC CA3'

Internal probe 5'GAG GCC ATC ACA TTG TAG CCC TCT GTG3'

hIL-17

Sense (5') 5' GTG AAG GCA GGA ATC ACA ATC 3'

Antisense (5') 5' ACC AGG ATC TCT TGC TGG AT 3'

Internal probe 5' CAG AGT TCA TGT GGT AGT CCA CGT TCC CA 3'

The DIG labelled PCR products were denaturated and hybridized with the biotinylated internal probe specifically designed to hybridize with each gene PCR product, and immobilized on streptavidin coated microtiter plates (3 hrs, 42°C), utilising the PCR ELISA DIG-detection kit (Roche). After washing, the bound PCR-products were incubated (30 min, 37°C) with an anti-DIG-horseradish peroxidase conjugated antibody followed by reaction (20 min) with the substrate 2,2'-azino-di(3-ethyl benzthiazoline sulfonate) (ABTS). The absorbance was then measured using spectrophotometry (see above, at 405/492 nm) in an ELISA plate reader (Labsystems Multiscan Multisoft, Vanda, Finland). The expression of transcripts for IL-17 mRNA was normalized to the expression of HPRT transcripts and shown as percent of the house-keeping gene (% HPRT). In parallel with tested samples, control samples for PCR, probe specificity, DNA contamination, hybridisation and sample dilution were run.

### Immunocytochemistry (ICC)

#### General procedure

BAL cells were fixed with formaldehyde (2%, 30 min, on ice) and washed twice in buffer prior to making cytospin preparations. Air-dried samples were stored frozen (-80°C) until further use. After thawing, samples were treated with donkey serum (10%) to avoid unspecific binding. Endogenous biotin was blocked using the Biotin Blocking System (DAKO corporation, Glostrup, Denmark).

#### IL-17A immunoreactivity (IR)

The intracellular expression of IL-17A protein was assessed utilising cytospin slides incubated with a polyclonal goat anti-human IL-17A antibody (R&D: 1 hr). As secondary antibody, a biotinylated F(ab')_2 _fragment donkey anti-goat IgG (Jackson ImmunoResearch laboratories Inc) was used, followed by alkaline phosphatase-conjugated streptavidin (DAKO). All the solutions above were supplemented with saponin (Sigma: 0.1 %), to permeabilise cell membrane. Bound antibodies were visualised with Vector Red Alkaline Substrate Kit (Vector Laboratories, Inc. Burlingame, CA, USA). Mayer's Hematoxylin (Sigma) was used for counterstaining.

#### MMP-9 immunoreactivity

The intracellular expression of MMP-9 protein was assessed utilising cytospin slides incubated with a polyclonal goat anti-human MMP-9 (R&D: 1 hr). The secondary antibody and the detection system were the same as for IL-17A (see above).

### Human neutrophilic lipocalin

The concentration of the neutrophil-specific activity marker human neutrophilic lipocalin (HNL) was measured in cell-free BAL fluid using a solid phase, double-ligand radioimmunoassay as described elsewhere [21].

### Data analyses

Data are presented as median (range) values unless otherwise stated. The analysis of differences between measurements prior to and after the exposure, were conducted utilizing the sign test, assuming binominal distribution. To avoid falsely positive conclusions, the significance level at each test was set to 0.05/3 (ie. 0.0167), since three key variables (IL-17A mRNA, IL-17 IR and MMP9 IR) were compared in the current material. *n *refers to number of human subjects.

## Results

### Clinical characteristics prior to exposure

Four males and 2 females with a median age of 22 (19–24) were included in the study. There were no apparent gender-related differences in pulmonary function of the subjects. FEV_1 _was 96 (92 – 105) % of predicted value and vital capacity 98 (90–102) % predicted value. All subjects had normal bronchial responsiveness to metacholine (PD_20_FEV_1 _for metacholine: 5.3 [1.7–15] mg).

### Cells in BAL fluid

The BAL recovery volume was similar before (195 [170–234] ml) and after (196 [190–212] ml) the exposure. The exposure caused more than a two-fold increase in the total BAL cell number, more than a five-fold increase in the number of lymphocytes and more than a ten-fold increase in the number of neutrophils.(Table 1).

**Table 1:** Concentration of BAL cells. BAL cells before and after exposure to organic dust in a swine confinement. Data are presented as 1 × 10^6^/L median (range).

**Cell type**	**Before**	**After**
Total cells	71,3 (57,9–80,9)	165,1 (98,2–365,6)
Lymphocytes	4,4 (1,2–10.0)	23,1 (9,8–61,6)
Neutrophils	2,3 (0,8–3,6)	23,1 (6,6–167,3)

### Messenger RNA for IL-17A

**Figure 1:**
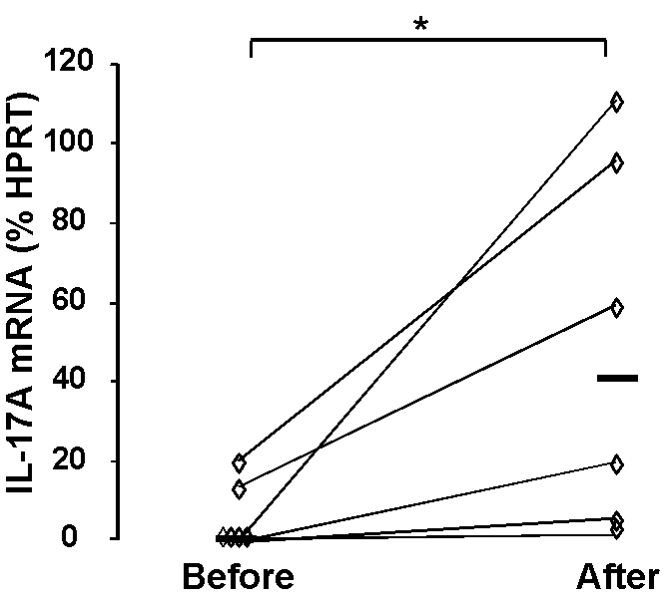
**Messenger RNA for IL-17A in BAL cells**. Messenger RNA for IL-17A in BAL cells (percentage of the house-keeping gene HPRT), measured 2 weeks before and 24 hours after exposure to organic dust in a swine confinement. Data are shown as individual (rhombs) plus median (bold horizontal lines) values. *: p < 0,0167; n = 6

Before the exposure, 4 out of 6 persons displayed no detectable levels of mRNA for IL-17A, whereas all six subjects displayed detectable and increased levels after the exposure (fig. 1).

### IL-17A immunoreactivity

**Figure 2:**
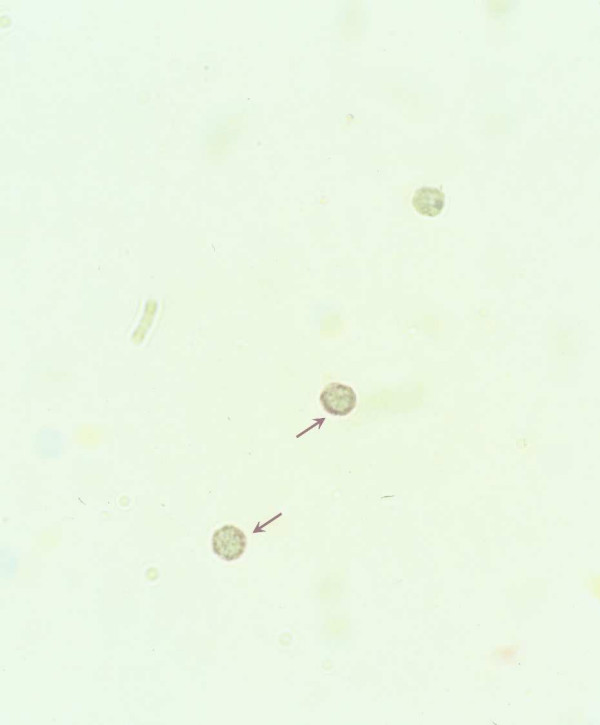
**Immunocytochemical detection of IL-17A protein in BAL lymphocytes**. BAL lymphocytes expressing IL-17A IR (brown arrows) after exposure to organic dust in a swine confinement, detected using immunocytochemistry.

**Figure 3:**
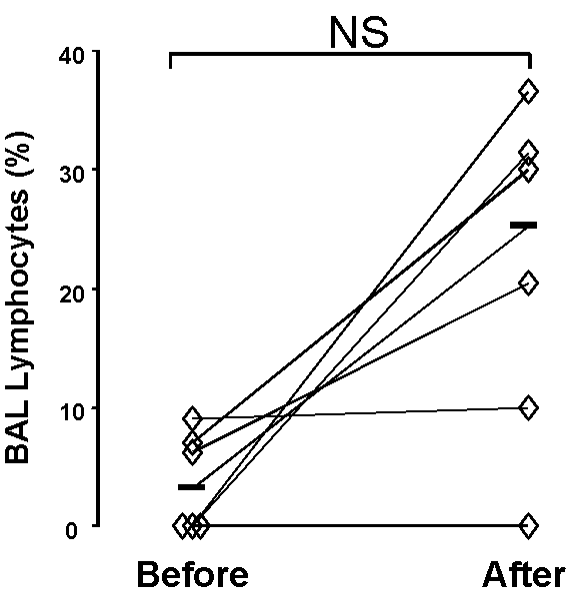
**BAL lymphocytes expressing IL-17A protein**. Percentage of BAL lymphocytes expressing IL-17A IR before and after the exposure to organic dust in a swine confinement. Data are shown as individual (rhombs) plus median (bold horizontal lines) values. NS: p > 0,0167; n = 6

IL-17A immunoreactivity was mainly detected in BAL lymphocytes (fig. 2). In addition, the percentage of lymphocytes positive for IL-17A immunoreactivity (fig. 3) tended to increase in 5 out of 6 subjects after the exposure. In contrast, a much lower fraction of macrophages expressed a weak signal for IL-17A immunoreactivity and there was no pronounced increase in this signal after the exposure (data not shown). BAL neutrophils did not display IL-17A immunoreactivity (data not shown)

### MMP-9 immunoreactivity

**Figure 4:**
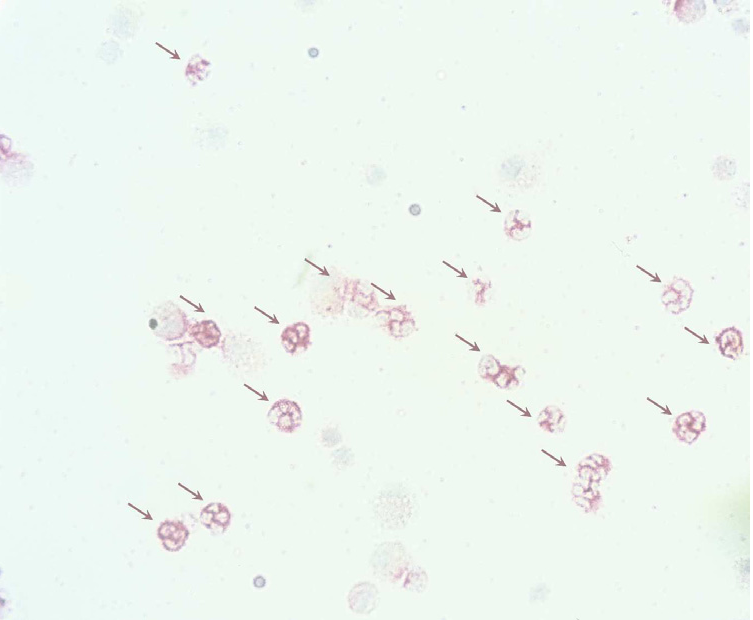
**Immunocytochemical detection of MMP-9 protein in BAL neutrophils**. Abundance of BAL neutrophils positive for MMP-9 IR (purple arrows) after exposure to organic dust in a swine confinement.

**Figure 5:**
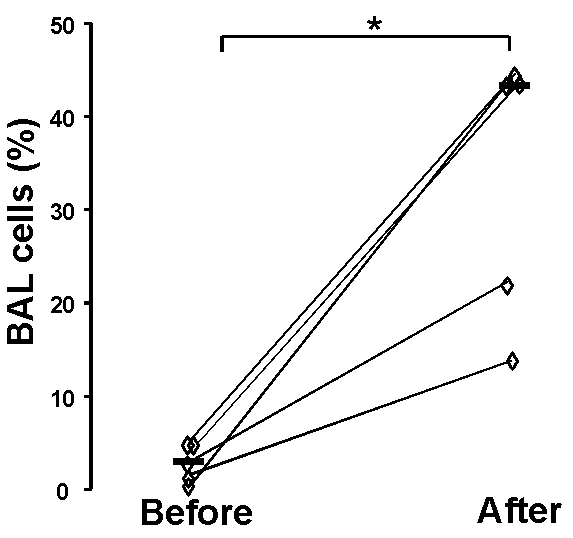
**BAL neutrophils expressing MMP-9 protein**. BAL neutrophils expressing MMP-9 IR (percent of total cells) before and after exposure to organic dust in a swine confinement. Data are shown as individual (rhombs) plus median (bold horizontal lines) values. *: p < 0,0167; n = 5.

Neutrophils were the prevailing cell type in BAL fluid expressing MMP-9 immunoreactivity (fig. 4) and this signal was substantially increased after exposure (fig. 5). However, the fraction of neutrophils (% all neutrophils) expressing MMP-9 immunoreactivity was not increased after exposure (data not shown). Very few macrophages expressed MMP-9 immunoreactivity and this signal was consistently weak (data not shown).

### HNL

The exposure did not cause any substantial increase in the concentration of free, soluble HNL in the BAL fluid, even though there was a weak average trend in this direction (data not shown). Furthermore, the amount of HNL per neutrophil tended to be decreased after exposure (data not shown).

## Discussion

The current study demonstrates that, in previously healthy human volunteers, exposure to organic dust substantially increases the messenger RNA for IL-17A in BAL cells. Our study also demonstrates that lymphocytes constitute the predominant cell type that expresses intracellular IL-17A protein among BAL cells. The demonstrated increase in IL-17A mRNA is associated with an increased absolute number of MMP-9 expressing BAL neutrophils at the same time point.

The aerosolised organic dust in a swine confinement, that was utilised as a pro-inflammatory stimulus in our study, is a more complex stimulus than endotoxin, because of its mixed content of grain dust, ammonia, fungi, Gram-positive and Gram-negative bacteria [15]. It has previously been shown that exposure to this type of organic dust causes severe inflammation in the bronchoalveolar space of humans [13,15,16]. Our current study now adds to these previous studies novel evidence of *de novo *synthesis of IL-17A by local cells within the bronchoalveolar space of humans [1]. Furthermore, our study forwards lymphocytes as a local source of IL-17A protein in the human bronchoalveolar space, because the intracellular immunoreactivity for this specific protein was found mainly in certain BAL lymphocytes. Noteworthy, even though this immunoreactivity should be regarded as a qualitative assessment primarily, it was increased eight-fold by average after exposure with only one subject lacking a detectable increase. Clearly, this is compatible with lymphocytes accounting for production of IL-17A protein in the bronchoalveolar space of humans. Indeed, our current results are supported by our previous demonstration of an increase in the final consequence of the increased transcription and intracellular loading of protein, namely in free, soluble IL-17A protein in cell-free BAL fluid [13]. It is also noteworthy that in our current study, certain but not all lymphocytes in the human bronchoalveolar space express IL-17A protein after exposure to organic dust. This observation is compatible with previous studies on activated mouse CD4^+ ^and CD8^+ ^lymphocytes from BAL and spleen as well as human CD4^+ ^and CD8^+ ^lymphocytes from blood [1,22-28]. Taken together, these observations warrant new specific cellular targets for additional analysis in future studies on human airways.

We assessed intracellular immunoreactivity for MMP-9 as a marker of proteolytic burden, both before and after exposure to organic dust. Even though some of the BAL macrophages displayed a very weak expression of MMP-9 immunoreactivity, as has previously been reported in a different inflammatory setting [29], we found the most pronounced increase in and expression of MMP-9 immunoreactivity consistently among BAL neutrophils. This observation fully supports that neutrophils can constitute an important source of MMP-9 in the human bronchoalveolar space *in vivo*, at least after exposure to organic dust. At the same time point after the exposure, the MMP-9 expression in neutrophils was associated with an increase in mRNA for IL-17A, even though the fraction (percentage) of neutrophils expressing MMP-9 was not increased. This is compatible with IL-17A indirectly contributing to the local proteolytic load, mainly through its increasing effect on the number of neutrophils within the human bronchoalveolar space; a type of mechanism supported by previous studies on the bronchoalveolar space of mice *in vivo *[9,12].

We also measured the concentration of free, soluble HNL protein in BAL fluid, as an assessment of neutrophil-specific activity [30]. However, we detected only a weak average trend towards a modest increase in the HNL concentration after exposure to organic dust and this increase did not even correspond to the substantial increase in neutrophil number; the amount of HNL per neutrophil was actually decreased after the exposure. Furthermore some subjects displayed even a decrease in the total HNL concentration after exposure. Moreover, separate and preliminary assessments of the myeloperoxidase (MPO) concentration in BAL fluid displayed a weak increase not corresponding to the increase in neutrophil number (data not shown). Similar findings on MPO and neutrophils have previously been published [31]. Taken together, all these findings imply that, in terms of MPO and HNL, there is actually no true increase in the average activity of each accumulated neutrophil in the human bronchoalveolar space after exposure to organic dust. Again, from a mechanistical point of view, this finding in the bronchoalveolar space of humans is supported by our recently published findings on the effect of recombinant IL-17A in the bronchoalveolar space of mice *in vivo *[12]. This IL-17A induces an increase of MMP-9 protein and in corresponding gelatinase activity, that is due to an increased number of neutrophils rather than an increased amount of MMP-9 per neutrophil [12].

In conclusion, supported by our recent study showing a corresponding increase in free, soluble IL-17A protein [13], the current study adds novel evidence for organic dust inducing *de novo *synthesis of IL-17A protein in a subset of local lymphocytes within the human bronchoalveolar space. Our current study on humans also adds evidence compatible with induced *de novo *synthesis of IL-17A being associated with an increased proteolytic burden due to a local accumulation of neutrophils rather than an increased activity in each of these inflammatory cells. Interventional studies will be required to determine whether targeting IL-17A is therapeutically beneficial in lung diseases characterised by excessive accumulation of neutrophils.

## Authors' contributions

LP, KL, AL – study design and coordination

SI, LP, KL, PV – exposure in swine farm, laboratory work

SI, AL – data analysis and interpretation of results

SI, LP, KL, PV, AL – preparation and revision of the manuscript
